# “*Wherever doctors cannot reach, the sunshine can*”: overcoming potential barriers to malaria elimination interventions in Haiti

**DOI:** 10.1186/s12936-018-2553-5

**Published:** 2018-10-29

**Authors:** Thomas Druetz, Katherine Andrinopoulos, Louis-Marie Boulos, Michaelle Boulos, Gregory S. Noland, Luccene Desir, Jean Frantz Lemoine, Thomas P. Eisele

**Affiliations:** 1 Department of Social and Preventive Medicine, University of Montreal School of Public Health, CP 6128, Succursale Centre-Ville, Montreal, QC H3C3J7 Canada; 2 Institut de Recherche en Santé Publique de l’Université de Montréal, Montreal, Canada; 3 Center for Applied Malaria Research and Evaluation, Department of Tropical Medicine, School of Public Health and Tropical Medicine, Tulane University, New Orleans, USA; 4 Department of Global Community Health and Behavioral Sciences, Tulane University, New Orleans, USA; 5 Centre d’Évaluation et de Recherche Appliquée, Port-au-Prince, Haiti; 6 The Carter Center, Atlanta, USA; 7 Ministry of Public Health and Population, Port-au-Prince, Haiti

## Abstract

**Background:**

Haiti and the Dominican Republic, the only two Caribbean countries with endemic malaria transmission, are committed to eliminating malaria. With a *Plasmodium falciparum* prevalence under 1% and a highly focal transmission, the efforts towards elimination in Haiti will include several community-based interventions that must be tailored to the local sociocultural context to increase their uptake. However, little is known about local community perceptions regarding malaria and the planned elimination interventions. The aim of this study is to develop a robust understanding of how to tailor, implement and promote malaria elimination strategies in Haiti.

**Methods:**

A cross-sectional qualitative study was conducted December 2015–August 2016 in Grande-Anse and the North Department in Haiti. Data collection included key informant interviews (n = 51), in-depth interviews (n = 15) and focus group discussions (n = 14) with health workers, traditional healers, teachers, priests or pastors, informal community leaders, public officials, and community members. Following a grounded theory approach, transcripts were coded and analysed using content analysis. Coded text was sorted by the types of interventions under consideration by the malaria elimination programme.

**Results:**

The level of knowledge about malaria was low. Many participants noted community beliefs about malaria being caused by magical phenomena in addition to vector-borne transmission. Participants described malaria as a problem rooted in the environment, with vector control the most noted method of prevention. Though participants noted malaria a severe disease, it ranked lower than other health problems perceived as more acute. Access barriers to healthcare were described including a lack of bed nets. Some distrust about pills, tests, and foreigners in general was expressed, and in few cases linked to previous experience with malaria campaigns under dictatorial regimes.

**Conclusions:**

There are several potential barriers and opportunities to implement community-based malaria elimination interventions in rural Haiti. Elimination efforts should include the collaboration of voodoo priests and other traditional healers, be coupled with solutions to wider community concerns or other health interventions, and learn from previous or similar programmes, such as the campaign to eliminate lymphatic filariasis. It is essential to engage with communities and gain their trust to successfully implement targeted aggressive elimination activities.

**Electronic supplementary material:**

The online version of this article (10.1186/s12936-018-2553-5) contains supplementary material, which is available to authorized users.

## Background

The island of Hispaniola (Haiti and Dominican Republic) is the only place in the Caribbean with endemic malaria transmission [1]. Haiti accounts for almost all malaria on the island, with 97% of the 22,949 confirmed cases for Hispaniola in 2016 located in Haiti [2]. Malaria infections are predominantly caused by *Plasmodium falciparum*, while *Plasmodium vivax* and *Plasmodium malariae* infections have been sporadically reported, but are most likely imported [3–5]. Overall, *P. falciparum* transmission is low, with repeated national-level household surveys in Haiti demonstrating a PCR-based parasite prevalence of 0.3–0.5% in 2011, 2012 and 2015 [4, 6]. Malaria transmission in Haiti is highly focal [1, 7, 8] and the incidence of falciparum cases coincides to some extent with the bimodal rainy season, usually resulting in two transmission peaks (November–January and May–June) [9].

The primary vector is presumably *Anopheles albimanus*, which is the only confirmed vector and the only species reported in the country over the last decade [10]. Despite *P. falciparum* endemicity in Haiti, there is no evidence of in vivo clinical resistance of the parasite to chloroquine, which is still used as a first-line treatment for uncomplicated malaria [11–16]. In addition, studies have found no resistance of *An. albimanus* to pyrethroids; resistance to dieldrin and fenitrothion is thought to be low, and only resistance to DDT is considered to be widespread [10].

These distinctive characteristics, along with the threat of emerging resistance to chloroquine [17] and renewed international attention towards shrinking the world malaria map [18], have led the Haitian and Dominican governments to commit to eliminating malaria by 2020 [19]. Over the last few years, several malaria prevention and diagnosis measures were adopted by Haiti’s Ministry of Public Health and Population, notably: adoption of rapid diagnostic tests (RDTs), drug-resistance monitoring, addition of primaquine to chloroquine as first-line therapy, establishment of an insectary and insecticide-resistance monitoring, implementation of surveillance sentinel sites, and strengthening of molecular and serological testing capacity at the national public health laboratory [20].

Further efforts towards the elimination of malaria in Haiti are planned and will include scale-up of community-based interventions, e.g., sensitization about malaria, distribution of long-lasting insecticidal nets (LLINs), larval habitat management, expansion of access to diagnosis and treatment with community case management, mass test and treat (MTaT) campaigns, and potentially other aggressive interventions, including targeted mass drug administration (tMDA) and indoor-residual spraying (IRS), in selected high transmission areas [3, 19].

Numerous studies have established the importance of tailoring community-based elimination interventions to the local sociocultural context to increase their effectiveness and uptake [21, 22]. However, very little is known about community perceptions in Haiti regarding malaria in general, and these community-based interventions in particular—an evidence gap that has recently been acknowledged in the literature [19, 23, 24]. The goal of this study was to develop a robust understanding of how to tailor, implement and promote malaria elimination strategies that could be considered by the Haitian Ministry of Health and its partners. The specific objectives were to: (1) assess community perceptions about malaria, including its relative importance and its risk factors, and (2) describe perceptions and acceptability of potential malaria elimination interventions.

## Methods

### Study sites

**Fig. 1:**
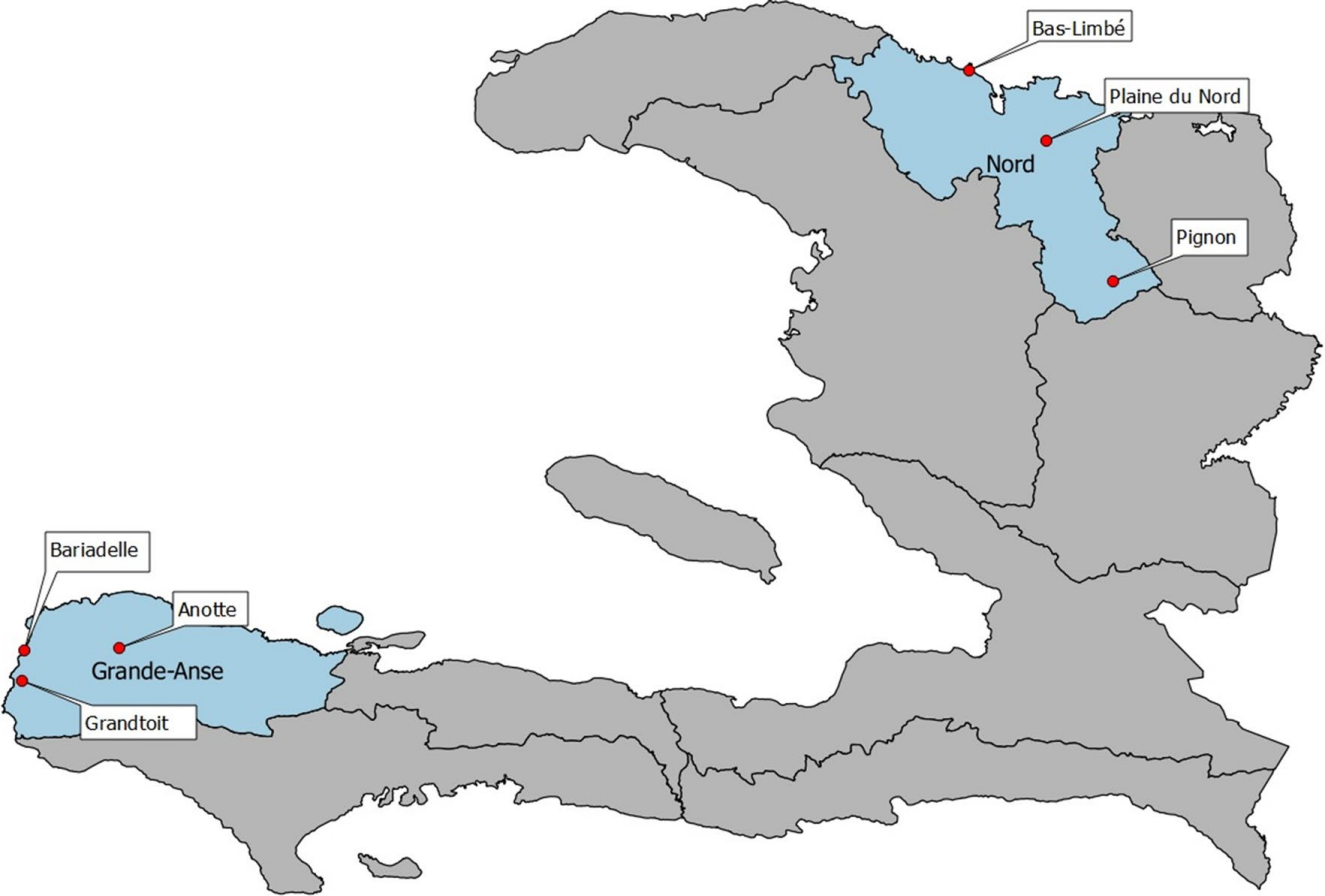
Study sites in Haiti

This cross-sectional qualitative study was conducted in two Haitian departments: Grande-Anse and the North Department. These were selected in collaboration with the National Programme against Malaria in Haiti because of their contrasting epidemiological profiles. Grande-Anse is located in the South-West peninsula and is the department with the highest malaria incidence in the country: 18.1 per 1000 inhabitants (total population: 468,301 [25]). In 2017, 8322 malaria cases were confirmed in Grande-Anse, out of a total of 17,878 nationwide. In the North Department, which includes the second largest city in Haiti (Cap-Haitien), only 67 confirmed malaria cases were reported out of a population of 1 million [25], which corresponds to an incidence rate < 0.1 per 1000 inhabitants. In each department, three study sites were selected for their heterogeneity regarding the environment, the level of urbanization, and the main socioeconomic characteristics of the local population (Fig. 1).

### Sampling and data collection

This qualitative study included key informant interviews in-depth interviews and focus group discussions. Participants for the interviews and focus groups were purposively recruited and included local healthcare workers, community health agents, community and religious leaders (teachers, public officials, informal leaders, and Catholic priests), traditional healers, voodoo priests (known as *hougans* in Haiti), and members of the communities. A total of 162 participants were recruited: 66 for individual interviews and 96 for focus group discussions. Participants were unknown to the research team members. Data saturation was discussed within the research team and taken into consideration to decide when stopping recruitment.

Two highly experienced qualitative researchers—one male, one female—performed the interviews and facilitated group discussions (MB and LMB). They were helped by two assistants who took field notes. The researchers and assistants introduced themselves and the organization they were working from. Guides were developed to structure interviews and focus group discussions. The guides were tailored to each type of participant and included open-ended questions to investigate the two main research objectives. An iterative approach was used so that information learned in earlier phases of the study informed sampling and questions in subsequent rounds of data collection. Interviews usually lasted between 45 and 90 min, and group discussions between 60 and 120 min. Data collection was completed between December 2015 and August 2016. Interviews were conducted privately at the office of the participants (including health facility, school, home, church) and were not repeated. Focus groups took place in private rooms located in public venues, most of the time a health facility or a school. Preliminary findings were discussed with participants at dissemination meetings.

### Key informant interviews

Key informant interviews were conducted with a total of 51 individuals, including departmental health authorities (n = 4), health workers (n = 7), teachers (n = 5), priests or pastors (n = 5), public officials (n = 8), traditional healers (n = 11) and matrons (n = 11). In most of the six study sites, all categories of key informant individuals were found and were available for an interview. No one refused to participate. Because data saturation was not achieved, the number of interviews with matrons and traditional healers is higher than for the other categories. An interview guide is attached in Additional file 1.

### In-depth interviews

Fifteen individual interviews were conducted in the six study sites with community members (n = 9) or informal community leaders or representatives (n = 6). These participants were either conveniently recruited after passing-by the study team, or they were referred during the key informant interviews or focus group discussions. All community members and informal leaders must have been living in their community for several years. There was at least one in-depth interview with a community member per study site. An interview guide is attached in Additional file 2.

### Focus group discussions

A total of 14 focus group discussions were animated with community health workers (n = 5), male community members (n = 4) and female community members (n = 5). There were seven participants per group discussion on average, for a total of 96 participants. Participants were purposefully selected to capture perspective of key groups as well as potential variation in perspectives within each group. Group discussions with community members were organized by age of the participants (18–35 years; 35 years and older). A guide to facilitate focus group discussions is attached in Additional file 3.

### Analyses

Interviews were audio-recorded, translated from Haitian Creole into English, and then transcribed. Final transcriptions and translations were checked by a second source and compared against the original recordings. Using a grounded theory approach, codes were developed a priori based on the research objectives and the community-based interventions that are considered in Haiti to eliminate malaria [26]. Preliminary interpretations were derived from the review of key transcripts and feedback from the data collection team. Codes were used to sort the data and allow for a review of related segments of text. Coding was mostly performed by one author (KA), but a sample of the material was coded by a second author (TD) to assess reliability (> 95%). Content analysis was used to synthesize themes, classify them by domain of community-based interventions, and examine variation across study sites and participant groups [27, 28]. Transcripts were coded using QSR Nvivo version 10. They were not returned to the participants.

A COREQ checklist (consolidated criteria for reporting qualitative research) is available in Additional file 4.

### Ethics

The study received approval from the Biomedical Institutional Review Board of Tulane University and from the National Committee of Bioethics in Haiti. Participants were informed of the study’s rationale and objectives, its procedures and its potential risks and benefits. They were informed of their right to refuse to participate, to drop out any time and to skip questions. Procedures to guarantee anonymity and confidentiality were explained. Verbal consent was obtained for all interviews and focus group participants.

## Results

A total of 162 participants were recruited for the interviews (n = 66) and focus group discussions (n = 96, in 14 groups). Details on participation by department and type of participants are displayed in Table 1. During the analysis, their perceptions were categorized according to the five different domains of community-based interventions that are considered in Haiti to eliminate malaria: knowledge and perceptions about malaria as a health issue, treatment-seeking practices, MTaT campaigns, vector control measures, and tMDA. The material was classified according to these domains in order to better inform decision-makers about the strategies relevant to them. By presenting results by domain of intervention, the intention is to help translating the evidence into practice, rather than to suggest that perceptions were intrinsically separated in the participants.

**Table 1:** Participants to the interviews and focus group discussions

Type of participant	Number of participants	
	Grande-Anse	North
Interviews		
Community leader or public official	8	9
Priest/Pastor	3	2
Traditional healer/Voodoo priest	3	8
Community members (male)	2	1
Community members (female)	3	3
Teacher	2	3
Health worker	4	3
Matrons (traditional birth attendant)	8	3
Departmental health authorities	2	2

	Number of groups	
Focus group discussions		
Community health agents	3	2
Community members (male)	2	2
Community members (female)	3	2

### Knowledge and perceptions about malaria as a health issue

Interviews and group discussions suggested that health issues are not always considered natural diseases in these communities; sometimes they are perceived as manifestations of magical powers, curses, or the devil. Malaria is particularly prone to this phenomenon because of its non-specific symptomatology: (1) its main symptom (fever) is common and caused by a variety of conditions; (2) symptoms can simultaneously affect different body entities (fever, vomiting, weakness, headaches, diarrhea, chills, etc.); and (3) it sometimes causes delirium and seizures, which gives the impression of being demonized or “zombied”.

*“[…] they do not believe in disease. They think that the fever is diabolic.”* (male community health agent)

*“The biggest challenge I face is that people don’t want to believe they have malaria. They always think someone is cursing them, that it’s fetishism, or a curse, or something in their diet.”* (female nurse)

*“You can also recognize malaria by a lot of symptoms like fever, delirium, the sick person can say that he saw the devil, he can say something else. You might think they’re possessed, because they stop making sense, but it’s actually the fever causing this. Malaria is making him delirious*.” (male community member)

Malaria was not considered the most important health issue by the participants. For the community members, social determinants of disease (poverty, lack of infrastructure and potable water, poor education and hygiene) were more important than any single health issue. Malaria was also perceived as being less severe or urgent compared to other diseases, especially typhoid fever and cholera.

*“[…] many Haitians will not take the disease seriously until it causes death.”* (male community member)

“*They are worried about cholera more than they are about malaria because malaria does not kill fast.”* (male community leader)

Participants frequently associated malaria with filthy environments like dirty yards, trash, swamps, and proximity to livestock. But interviews also revealed that there was no clearly defined high-risk population; everyone was considered at risk because mosquitoes are everywhere and bite indiscriminately. Regarding its causes, malaria was considered a community threat, not an individual disease. There was no indication of community stigma for having malaria.

*“I can confidently say I will never get AIDS, you see? But I can’t say that I will never catch malaria. Because mosquitoes are everywhere. They can bite you wherever you are, in your sleep, outside. There are some diseases I know I can never get, but malaria isn’t one of those. As long as malaria isn’t eliminated in the entire country, everyone is at risk of getting it someday.”* (male traditional healer)

*“There is no distinction between the rich and the poor, the ugly and the beautiful, mosquitoes bite everyone and if you’re bitten by an infected mosquito, you will be infected too.”* (male community member)

### Treatment-seeking practices

Participants reported during the interviews that seeking treatment for fever at a health facility is not common and is often delayed. It is common to visit health facilities only when symptoms worsen. Four main barriers to prompt treatment-seeking were identified: (1) distance to the health facility; (2) costs associated with the consultation; (3) the relative non-urgent perception of malaria compared to other diseases, such as cholera; and (4) beliefs in the non-natural origin of diseases in general, and of malaria in particular.

“*But with malaria, the person is more likely to stay in the community and let the disease progress, drink tea. By the time the patient gets here, they’re already in a coma due to cerebral malaria and there’s nothing we can do.”* (female nurse)

The qualitative analysis revealed that different treatment-seeking patterns exist among those with a fever, but usually the first action consists in “self-medication” at home, e.g., drinking certain kinds of tea. If the fever persists and the person seeks treatment outside of the home, different options for receiving healthcare exist, including pharmacists at drug stores, *doktè fey* (herbalists), voodoo priests (*hougan*, also known as *boko*, *mambo*, or *gangan*), and health personnel at health facilities. At the time of the study, community health workers were present in some communities but were not allowed by the Ministry of Health to test or provide treatment for malaria. Instead, they intervened preventively by disseminating health messages, increasing awareness about malaria and other health issues, and encouraging community members to seek treatment at health facilities in case of fever.

*“You have people that know that if they have a fever it’s because there is something wrong with the body and at that point they will report to a doctor. Others may think that it’s something they caught in the street and will want to seek help from a traditional healer/herbalist. Meanwhile you have other people who just believe it’s some supernatural disease therefore they will take a spiritual healer to help them or they have to pray.”* (female community member)

Seeking treatment from different providers was reported to be common. In particular, a febrile person may consult a *hougan* in the community, and if he/she is not cured, they may then go to a health facility. Most of the *hougan* who were interviewed stated that they voluntarily refer patients with fever and/or severe symptoms to a nurse or a doctor, especially if their own therapy does not work. However, there is no formal relationship between health facilities and *hougan* who are active in their communities. Community health workers encourage people with suspected malaria to visit health facilities, but acknowledge that *hougan*, who are well-known and respected in the communities, could serve as an additional convenient outlet for diagnosis and treatment if they were trained to manage uncomplicated malaria.

*“This is a Haitian problem. Haitians have this inner myth: diseases are unnatural. Whenever they fall sick, they seek treatment elsewhere, [at] hougan. Hospitals are not the primary source of seeking treatment. If the hougan does not work, they then decide to consult a doctor. So this inner myth leads them to the wrong path of seeking treatment first.”* (male community health agent)

*“They [the hougan] are leaders too because a lot of people go to see them. The MSPP [Ministry of Health] must work with them too; they* *must train them as well”.* (female community health agent)

### Mass test and treat

Malaria rapid diagnostic tests are commonly used at health facilities, and health personnel have not experienced resistance to their use. The idea of organizing free malaria screening campaigns in these communities was mostly welcomed by the participants in in-depth interviews and focus groups. However, participants also mentioned that mistrust from some community members would be a challenge. Some may fear that their blood will be used for other purposes (HIV testing, black magic, etc.). Interviews also revealed that people without symptoms may be more likely to avoid screening stations or to refuse blood tests at their homes.

*“Sometimes people can refuse to be tested because they are not sure that the reasons put forward by the one giving it are the real ones and they fear that they could be tested for any kind of weird disease without their knowledge and consent*.” (female traditional birth attendant)

*“If you have to take a blood sample, the patient might think that you are going to do an HIV test for him although you said malaria test.”* (male community leader)

*“Last time something like that took place, there are people that said that they would not be willing to give their blood because they didn’t know what they would do with the blood that was collected.”* (female community member)

While participants argued that a door-to-door approach would optimize coverage of malaria screening campaigns, they emphasized that any such campaign should be official to overcome mistrust from the community. Participation would likely increase if tests were performed by well-known health personnel (not foreigners) with the support of community leaders. If fixed stations are used, testing and treating have to be performed by health personnel in official venues (such as dispensaries or schools).

*“If they see a stranger come into do these tests, they might think they’re trying to get money from them. But if a nurse or a healthcare worker from the health center, someone they know, administers the test, they’ll be more inclined to trust them*.” (female nurse, #15)

### Vector control

Community members interviewed reported that they appreciated LLINs as a protective measure against malaria, but indicated that they are too expensive (~ 5 USD) to buy, and that mass distribution campaigns often miss hard-to-reach households or do not distribute enough LLINs per household. Also, they are not easy to install and only partially reduce nuisance biting, because mosquitoes other than *An. albimanus* are abundant and bite during the daytime as well.

*“It’s more the lack of financial means that prevent people from buying nets than the knowledge of its importance*.” (female community member)

*“Because mosquitoes do not only bite at night so even if you are sleeping under a bed net, during the late afternoons you can get bitten*.” (male community leader)

During the interviews, several participants described the environment as the underlying cause of malaria, and stated that it was essential to consider working “upstream” and to eliminate vector breeding sites. An unclean environment was perceived as the main reason for putting everyone at risk.

*“What is important is to go to the root of the problem. As long as there is stagnant water around, old tires, coconut husks, mosquitoes will always multiply. Even if people come to treat you, you will still have malaria, because the source of the problem is those breeding sites*.” (male community member)

### Mass drug administration

While most of the participants said they would support MDA campaigns for malaria, they also suspected that the uptake in the communities would likely be an issue, and that sensitization would be essential. Even then, convincing people to accept malaria treatment in the absence of symptoms or a proper diagnosis will be a challenge according to some interviewees. Mistrust about pills freely distributed is to be expected, especially among those who believe in fetishism. Fear of side effects and reluctance to take several pills on an empty stomach were issues recently experienced by the Ministry of Health during malaria outbreak responses. While most of the participants anticipated that some community members would not take drugs during an MDA campaign, no consensus emerged about who or what groups would most likely resist, nor what the best option would be to increase participation in the programme.

*“I will not take pills against malaria without being diagnosed with the disease*.” (female community member)

*“Sometimes people say they have not eaten anything yet and this is a problem because they can’t take it [malaria treatment] on an empty stomach*.” (female doctor)

The similarities with the ongoing drug distribution campaign for lymphatic filariasis in Haiti may provide an avenue for encouraging community members to accept MDA for malaria. Older community members also associated MDA with the activities of the *Service National d’Éradication de la Malaria* (SNEM, the eradication programme of the 1960s–1970s), and emphasized the importance of changing the mode of distribution and no longer forcing people to take anti-malarial pills.

*“They will all accept [MDA] because they all have had this experience before with the filariasis programme and all the community people in this area took the drugs without any fuss […]”.* (male community leader)

*“I remember when I was a kid, people were talking about SNEM. They said some people went to hide to avoid taking the drugs, but the health agents or Tonton makout [Duvalier’s militia force] ran after them to force them to take medications. Today the Haitian government can no longer force people but can motivate people to explain how the drug can help people have a better life*.” (male teacher)

### Box 1 Recommendations for community-based malaria elimination interventions in Haiti

- Increase community awareness of malaria, its symptoms, and the importance of seeking prompt treatment in case of fever.
- Settle with this pluralistic health system, particularly the presence of voodoo priests in the communities.
- Engage with and gain trust from the communities before launching mass campaigns of blood tests or drug administration.
- Prioritize public health interventions over actions targeting sub-populations.
- Couple malaria elimination efforts with solutions to wider community concerns, distal environmental factors or other health interventions that are seen as a higher priority.
- Learn from previous experiences and decentralize the implementation of malaria elimination activities.

## Discussion

To our knowledge, this is the first study conducted in Haiti which assesses community perceptions about potential interventions to eliminate malaria. Results suggest that several obstacles and opportunities can influence the effectiveness of community-based malaria interventions in Haiti, and arguably should be taken into consideration in tailoring the elimination strategy to the local context. Key recommendations are summarized in Box 1.

Knowledge about malaria and concern about this health issue was low among community members, and this may serve as a challenge to elimination goals in Haiti. Malaria was systematically not considered by the participants as important or as urgent as other health issues. This can be partly due to the fact that overall malaria incidence has been low for several decades in Haiti [3]. Also, it is noteworthy that during a survey that we recently administered to 2500 inhabitants of the Grande-Anse Department (Haiti), only a third of the participants were even familiar with the term “malaria” (results to be published). This is lower than measures obtained in other elimination settings, such as Sri Lanka or Swaziland, where knowledge about malaria was nearly universal at 99% and 93%, respectively [29, 30]. This study also suggests that treatment for fever is rarely sought through the formal health sector, or is sought with a significant delay. Similar to conclusions from a previous study in Haiti, results from this investigation suggest that it is essential to increase community awareness of malaria, its symptoms, and the importance of seeking prompt treatment [31].

This study corroborates previous findings about the use of multiple healing systems in Haiti for health problems including allopathic, homeopathic, and spiritual remedies [32]. Beliefs supporting modern and traditional medicine coexist within communities and individuals [33]. While the predominant use of *hougans* and herbalists has been demonstrated in treatment-seeking practices of people afflicted with HIV, mental illness or lymphatic filariasis in Haiti, this study reveals that similar patterns exist in febrile individuals [34–37]. At the same time, using *hougans* remains stigmatized, and few participants acknowledged having visited one themselves. Because of its symptomatology, malaria is particularly likely to be interpreted as the expression of magical, supernatural phenomena. Malaria elimination efforts must address the pluralistic health system, particularly the presence of voodoo priests in the communities who are often the first place people seek care. These individuals could be instrumental in referring febrile individuals to health facilities and community health workers, as has been observed elsewhere [38–41]. As malaria transmission decreases and becomes more clustered in residual pockets, *hougans* could also be used to rapidly inform health authorities of any new febrile patient, which could trigger a foci investigation. In this context, involving traditional medicine practitioners will be an essential component for malaria elimination programmes [42]. Different types of incentives can be used to increase their willingness to participate, e.g. organizing training sessions to improve their competence or inviting them to introduce health personnel to herbal medicine [40].

Community case management, where community health workers provide malaria diagnosis and treatment in their communities, as well as potentially MTaT, appear to be promising strategies in the Haitian context because they have the potential to mitigate three of the most commonly reported barriers to accessing treatment at health facilities: distance to the health centre, costs associated with the visit, and the perceived non-urgency of the disease. It is revealing that nearly all health facilities that were visited during the study charged administrative fees for malaria testing and/or treatment, although they should be entirely free according to a national policy.

Improving access to malaria testing and treatments can contribute to reducing malaria transmission and preventing severe disease and deaths [3, 43]. But to optimize their potential, it appears essential to gain the trust of communities, notably by guaranteeing that their blood will only be used for malaria testing and not for HIV testing or for magic. Performing blood tests in official local venues, and/or with the participation of local health authorities known in the community, was suggested by several participants. This is aligned with previous studies suggesting general distrust in the communities of foreigners and non-governmental organizations working in the health sector [44].

Malaria was perceived by most community members as an indiscriminate, environmental disease. Malaria is not a taboo subject and infected people are not stigmatized. As a consequence, public health interventions could be more widely accepted than interventions targeting specific subpopulations like high-risk groups or symptomatic persons. This is of high relevance in elimination settings since public health interventions are required to attack the entire parasite reservoir, irrespective of symptomology [45]. Secondly, the elimination strategy will likely receive more community support if it entails actions on more distal, environmental determinants of malaria, such as elimination of larval sites, drainage of swamps, waste management, or improvement in public infrastructure. As recommended previously, results from the present study suggest that coupling malaria elimination interventions with solutions to wider community concerns, or pairing them with other health interventions that are seen as a higher priority in the communities, would be beneficial [24].

Most participants recommended to increase access to LLINs. This is supported by results from previous studies that showed that only 59–68% of the households in Haiti possess at least 1 LLIN [8, 46, 47]. Universal coverage, defined as at least 1 LLIN per 2 household residents, is most likely below this range—it reached only 24% among the 2500 households recently surveyed in Grande-Anse (results to be published). While increasing access to LLINs seems to be a priority for the majority of the participants, some acknowledged that LLINs are of little help in reducing the mosquito population or the daily number of bites. In addition, while the evidence is scant, one study appears to suggest that their effectiveness to prevent malaria in Haiti is limited due to *An. albimanus* exophagic preferences [46].

Finally, previous experiences are likely to influence community perceptions of elimination interventions and should be taken into consideration [48, 49]. In particular, tMDA campaigns need to distance themselves from the Duvalier regime interventions, notably by obtaining individuals’ consent and local support. The lymphatic filariasis elimination programme introduced in 2000 is a recent example of a well-accepted tMDA strategy based on its ability to mobilize strong community engagement and support [50]. Decentralization can also be helpful by reducing the perception that interventions are driven or imposed by foreigners coming either from Port-au-Prince or other countries [21].

## Limitations

This study was conducted in only two departments of Haiti and may not be representative of the perceptions, knowledge, attitudes and practices of the entire population towards malaria elimination interventions. A Hawthorne effect cannot be ruled out and it is possible that a social desirability bias has affected the participants’ answers on sensitive topics [26]. To reduce this risk, individual interviews were performed by fieldworkers highly trained in qualitative research methods. In addition, in group discussions, community members were gathered with individuals of the same sex and age range (18–34 years; 35 years and older).

Also, this study does not claim to be exhaustive on potential elimination interventions, but focuses on those that are most likely to be implemented in Haiti. For example, indoor residual spraying, a strategy that has not been widely used in Haiti in the last few decades, was not specifically investigated here. While this study suggests that environmental actions are welcomed by community members, indoor residual spraying usually raise issues of privacy and security, as it requires removing all items from houses [51]. Additional research could be needed depending on the types of interventions that are implemented under the elimination strategy.

## Conclusions

Haiti and Dominican Republic are committed to eliminating malaria by 2020. In Haiti, where most of the malaria burden falls, health authorities and their partners are planning to implement or scale-up multiple community-based interventions. It is important to explore and consider the local perceptions about these interventions in order to increase their uptake and acceptability. This study identified several obstacles and opportunities at the community level, and formulated recommendations to help tailor and improve the effectiveness of malaria elimination strategies in Haiti. In particular, engaging with communities and gaining their trust seems to be critical to the successful implementation of targeted aggressive elimination activities.

## Additional files

**Additional file 1.** Interview guide for key informant interviews.

**Additional file 2.** Interview guide for in-depth interviews.

**Additional file 3.** Guide for focus group discussions.

**Additional file 4.** COREQ (consolidated criteria for reporting qualitative research) checklist.

## Abbreviations

EAG: easy access group
KAP: knowledge, attitudes and practices
LLIN: long-lasting insecticidal nets
MTaT: mass test and treat
RDT: rapid diagnostic test
SNEM: Service national d’éradication de la malaria
tMDA: targeted mass drug administration

## References

[1] Eisele TP, Keating J, Bennett A, Londono B, Johnson D, Lafontant C (2007). Prevalence of *Plasmodium falciparum* infection in rainy season, Artibonite Valley, Haiti, 2006. Emerg Infect Dis.

[2] WHO (2017). World malaria report 2017.

[3] CHAI (2013). The feasibility of malaria elimination on the island of Hispaniola, with a focus on Haiti: an assessment conducted January–June 2013.

[4] Lucchi NW, Karell MA, Journel I, Rogier E, Goldman I, Ljolje D (2014). PET-PCR method for the molecular detection of malaria parasites in a national malaria surveillance study in Haiti, 2011. Malar J.

[5] Lindo JF, Bryce JH, Ducasse MB, Howitt C, Barrett DM, Lorenzo Morales J (2007). *Plasmodium malariae* in Haitian refugees, Jamaica. Emerg Infect Dis.

[6] Haïti PSI (2011). Étude TRaC sur la possession et l’utilisation des moustiquaires imprégnées d’insecticides et la prévalence du paludisme en Haïti.

[7] Elbadry MA, Tagliamonte MS, Raccurt CP, Lemoine JF, Existe A, Boncy J (2017). Submicroscopic malaria infections in pregnant women from six departments in Haiti. Trop Med Int Health.

[8] Elbadry MA, Al-Khedery B, Tagliamonte MS, Yowell CA, Raccurt CP, Existe A (2015). High prevalence of asymptomatic malaria infections: a cross-sectional study in rural areas in six departments in Haiti. Malar J.

[9] Raccurt C (2004). Le point sur le paludisme en Haïti. Cahiers Santé.

[10] Frederick J, Saint Jean Y, Lemoine JF, Dotson EM, Mace KE, Chang M (2016). Malaria vector research and control in Haiti: a systematic review. Malar J.

[11] Neuberger A, Zhong K, Kain KC, Schwartz E (2012). Lack of evidence for chloroquine-resistant *Plasmodium falciparum* malaria, Leogane, Haiti. Emerg Infect Dis.

[12] Duverseau YT, Magloire R, Zevallos-Ipenza A, Rogers HM, Nguyen-Dinh P (1986). Monitoring of chloroquine sensitivity of *Plasmodium falciparum* in Haiti, 1981–1983. Am J Trop Med Hyg.

[13] Magloire R, Nguyen-Dinh P (1983). Chloroquine susceptibility of *Plasmodium falciparum* in Haiti. Bull World Health Organ.

[14] Bonnlander H, Rossignol AM, Rossignol PA (1994). Malaria in central Haiti: a hospital-based retrospective study, 1982–1986 and 1988–1991. Bull Pan Am Health Organ.

[15] Londono BL, Eisele TP, Keating J, Bennett A, Chattopadhyay C, Heyliger G (2009). Chloroquine-resistant haplotype *Plasmodium falciparum* parasites, Haiti. Emerg Infect Dis.

[16] Okech BA, Existe A, Romain JR, Memnon G, Victor YS, de Rochars MB (2015). Therapeutic efficacy of chloroquine for the treatment of uncomplicated *Plasmodium falciparum* in Haiti after many decades of its use. Am J Trop Med Hyg.

[17] Keating J, Krogstad DJ, Eisele TP (2010). Malaria elimination on Hispaniola. Lancet Infect Dis.

[18] Feachem RG, Phillips AA, Hwang J, Cotter C, Wielgosz B, Greenwood BM (2010). Shrinking the malaria map: progress and prospects. Lancet.

[19] Boncy PJ, Adrien P, Lemoine JF, Existe A, Henry PJ, Raccurt C (2015). Malaria elimination in Haiti by the year 2020: an achievable goal?. Malar J.

[20] Lemoine JF, Boncy J, Filler S, Kachur SP, Fitter D, Chang MA (2017). Haiti’s commitment to malaria elimination: progress in the face of challenges, 2010–2016. Am J Trop Med Hyg.

[21] Atkinson JA, Vallely A, Fitzgerald L, Whittaker M, Tanner M (2011). The architecture and effect of participation: a systematic review of community participation for communicable disease control and elimination. Implications for malaria elimination. Malar J.

[22] Whittaker M, Smith C (2015). Reimagining malaria: five reasons to strengthen community engagement in the lead up to malaria elimination. Malar J.

[23] Adrien P, Boncy J, Lemoine JF, Existe A, Juin S, Amouzou S (2016). Malaria elimination in Haiti: challenges, progress and solutions. Clin Microbiol Infect Dis.

[24] Bardosh K, Jean L, Beau De Rochars V, Lemoine J, Okech B, Ryan S (2017). A culturally competent approach to larval source reduction in the context of lymphatic filariasis and malaria elimination in Haiti. Trop Med Infect Dis.

[25] Ministère de l’Économie et des Finances (2015). Population totale de 18 ans et plus, Ménages et densités estimés en 2015.

[26] Miles MB, Huberman AM, Saldaña J (2014). Qualitative data analysis: a methods sourcebook.

[27] Patton MQ (2015). Qualitative research & evaluation methods: integrating theory and practice.

[28] WHO (2012). Community-based reduction of malaria transmission.

[29] Hlongwana KW, Mabaso ML, Kunene S, Govender D, Maharaj R (2009). Community knowledge, attitudes and practices (KAP) on malaria in Swaziland: a country earmarked for malaria elimination. Malar J.

[30] Kirkby K, Galappaththy GN, Kurinczuk JJ, Rajapakse S, Fernando SD (2013). Knowledge, attitudes and practices relevant to malaria elimination amongst resettled populations in a post-conflict district of northern Sri Lanka. Trans R Soc Trop Med Hyg.

[31] Keating J, Eisele TP, Bennett A, Johnson D, Macintyre K (2008). A description of malaria-related knowledge, perceptions, and practices in the Artibonite Valley of Haiti: implications for malaria control. Am J Trop Med Hyg.

[32] Miller NL (2000). Haitian ethnomedical systems and biomedical practitioners: directions for clinicians. J Transcult Nurs.

[33] Vonarx N (2011). Haitian vodou as a health care system: between magic, religion, and medicine. Altern Ther Health Med.

[34] Coreil J, Mayard G, Louis-Charles J, Addiss D (1998). Filarial elephantiasis among Haitian women: social context and behavioural factors in treatment. Trop Med Int Health.

[35] Farmer P (2006). AIDS and accusation: Haiti and the geography of blame.

[36] Desrosiers A, St Fleurose S (2002). Treating Haitian patients: key cultural aspects. Am J Psychother.

[37] Khoury NM, Kaiser BN, Keys HM, Brewster AR, Kohrt BA (2012). Explanatory models and mental health treatment: is vodou an obstacle to psychiatric treatment in rural Haiti?. Cult Med Psychiatry.

[38] Winch PJ, Gilroy KE, Wolfheim C, Starbuck ES, Young MW, Walker LD (2005). Intervention models for the management of children with signs of pneumonia or malaria by community health workers. Health Policy Plan.

[39] Falade C, Osowole O, Adeniyi J, Oladepo O, Oduola AM (2004). Attitude of health care workers to the involvement of alternative healthcare providers in the home management of childhood malaria. Int Q Commun Health Educ.

[40] Makundi EA, Malebo HM, Mhame P, Kitua AY, Warsame M (2006). Role of traditional healers in the management of severe malaria among children below five years of age: the case of Kilosa and Handeni Districts, Tanzania. Malar J.

[41] Gomes MF, Faiz MA, Gyapong JO, Warsame M, Agbenyega T, Babiker A (2009). Pre-referral rectal artesunate to prevent death and disability in severe malaria: a placebo-controlled trial. Lancet.

[42] Graz B, Kitua AY, Malebo HM (2011). To what extent can traditional medicine contribute a complementary or alternative solution to malaria control programmes?. Malar J.

[43] Druetz T, Fregonese F, Bado A, Millogo T, Kouanda S, Diabate S, Haddad S (2015). Abolishing fees at health centers in the context of community case management of malaria: what effects on treatment-seeking practices for febrile children in rural Burkina Faso?. PLoS ONE.

[44] Sethi S, Belliard JC (2009). Participatory health assessment in Haiti: practical tools for community empowerment. Prog Community Health Partnersh.

[45] Lindblade KA, Steinhardt L, Samuels A, Kachur SP, Slutsker L (2013). The silent threat: asymptomatic parasitemia and malaria transmission. Expert Rev Anti Infect Ther.

[46] Steinhardt LC, Jean YS, Impoinvil D, Mace KE, Wiegand R, Huber CS (2017). Effectiveness of insecticide-treated bednets in malaria prevention in Haiti: a case–control study. Lancet Glob Health.

[47] Stephenson CJ, Rossheim ME, Frankenfeld CL, Boncy J, Okech BA, von Fricken ME (2017). Cross-sectional analysis of the association between bedtime and malaria exposure in the Ouest and Sud-Est Departments of Haiti. Acta Trop.

[48] Druetz T, Kadio K, Haddad S, Kouanda S, Ridde V (2015). Do community health workers perceive mechanisms associated with the success of community case management of malaria? A qualitative study from Burkina Faso. Soc Sci Med.

[49] Bingham A, Gaspar F, Lancaster K, Conjera J, Collymore Y, Ba-Nguz A (2012). Community perceptions of malaria and vaccines in two districts of Mozambique. Malar J.

[50] Lemoine JF, Desormeaux AM, Monestime F, Fayette CR, Desir L, Direny AN (2016). Controlling neglected tropical diseases (NTDs) in Haiti: implementation strategies and evidence of their success. PLoS Negl Trop Dis.

[51] Ediau M, Babirye JN, Tumwesigye NM, Matovu JK, Machingaidze S, Okui O (2013). Community knowledge and perceptions about indoor residual spraying for malaria prevention in Soroti district, Uganda: a cross-sectional study. Malar J.

