# Supplementary material for: “Wherever doctors cannot reach, the sunshine can”: overcoming potential barriers to malaria elimination interventions in Haiti
Source: Malar J. 2018 Oct 29;17:393. doi: 10.1186/s12936-018-2553-5 (PMC6206681; doi:10.1186/s12936-018-2553-5)
Supplement: Supplementary file 1 — Additional file 1. Interview guide for key informant interviews. [file 12936_2018_2553_MOESM1_ESM.doc]

**GUIDE D’ENTREVUE HOUGANS / LEADERS COMMUNAUTAIRES**

**ET OFFICIELS GOUVERNEMENT**

INTERVIEW GUIDE HOUGANS / COMMUNITY LEADERS / GOVERNMENT OFFICIALS

**LES ÉTAPES DE L’ENTREVUE**

**THE STAGES OF THE INTERVIEW**

| **Etape 1. Présenter l'équipe de recherche : Modérateurs, preneurs de notes**  Step 1. Introduce the research team: Moderators and Note taker  **Etape 2. Présenter le sujet de recherche: Je vous remercie d'être venus à notre groupe de discussion sur le paludisme. Nous vous remercions d'être ici avec nous. Nous menons** cette entrevue **pour apprendre de votre expérience en tant qu’un important leader de la communauté. Nous sommes particulièrement intéressés à savoir plus au sujet de la communauté que vous servez et de vos expériences concernant le paludisme.**  Step 2. Present the research topic: Thank you for coming to our conversation on malaria. Thank you for being here with us. We conduct this interview to learn from your experience on malaria in your community. We are particularly interested to learn more about the community you are working with and your experiences on malaria.  **Etape 3. Règles du jeu : Avant de commencer, permettez-moi de passer en revue quelques notions de base sur la façon dont nous allons travailler. Il est important de se rappeler qu'il n'y a pas de bonnes ou de mauvaises réponses aux questions posées. Vous pouvez avoir des opinions différentes sur certains de ces sujets. Même si ce que vous avez à dire est différent de ce que pense les autres, nous voulons l'entendre. Nous voulons entendre ce que tout le monde pense. En outre, la conversation sera menée en respectant les règles suivantes: Chacun de vous aura la chance de s'exprimer en respectant le droit d'expression des autres. Vous n’ etes pas obligés d'être d'accord ou en désaccord avec l’autre suivant le cas et devrez présenter vos arguments pour appuyer votre point de vue. Vous pouv prendre la parole un seul à la fois et parler à haute et intelligible voix pour nous permettre d'entendre clairement et enregistrer ce que vous dites. Et enfin rappelez-vous que tous les commentaires ou opinions exprimés par chacun de vous sont considérés comme strictement confidentiels et nous vous demandons de ne pas les partager ou d'en discuter en laissant la table de réunion. Avez-vous des questions ou un point à éclaircir avant le début de la conversation.**  Step 3. Rules: Before we start, let me review some basics principles about how we will proceed. It is important to remember that there are no right or wrong answers to the questions. Each of you can have different opinions on some of these subjects. Even if what you say is different from what others think or say we want to hear it. In addition, this conversation will be conducted with the following rules: Each of you will have the opportunity to speak while respecting the right of expression of others. You are not obliged to agree or disagree with what is said as appropriate and must present your arguments to support your views. You can speak and talk in a loud and clear voice to allow us to clearly hear and record what you say.  And finally remember that any comments or opinions expressed are considered strictly confidential and we ask you not to share or discuss it outside after leaving the meeting table. Then I ask you if you have questions or point that need clarification before starting the discussion.  **Etape 4. Signature du consentement informé**  Step 4. Signature of informed consent forms.  **Etape 5. Identification des participants - Remise des badges**  Step 5. Identification of the participants - Badges  **Etape 6. Collecte des données démographiques**  Step 6. Demographic data collection  **Etape 7. Entrevue**  Srep 7. Interview |
| --- |

**SECTION 1. PRESENTATION (5 - 10 minutes)**

**1.1. PRESENTATION DES MODERATEUR ET PRENEUR DE NOTES**

1.1. PRESENTATION OF MODERATOR AND NOTES TAKERS

**1.2. DONNEES DEMOGRAPHIQUES**

1.2. DEMOGRAPHICS

**1.3. PRESENTATION DU OU DES PARTICIPANTS SUIVANT LE CAS**

1.3. PRESENTATION OF PARTICIPANTS

**A INVESTIGUER : 1.3.1.. PARLEZ-MOI DE VOUS ?**

PROBE :1.3.1. TELL ME ABOUT YOU?

**A INVESTIGUER : 1.3.2. ET DE VOS ACTIVITES AU NIVEAU DE VOTRE COMMUNAUTE ?**

PROBE :1.3.2. WHAT DO YOU DO IN THE COMMUNITY ?

**HOUGANS ET TRADIPRACTICIENS**

**FOR HOUGANS ET TRADIPRATICIENS**

**A INVESTIGUER : 1.3.3. PARLEZ-MOI DE VOTRE FONCTION DANS LA COMMUNAUTE ?**

PROBE :1.3.3. TELL ME ABOUT YOUR FUNCTIONS AS A HOUGAN IN THE COMMUNITY ?

YOUR FUNCTION AS A TRADITIONAL HEALER?

**A INVESTIGUER : 1.3.4 . QUAND VIENT-ON VOUS VOIR EN CAS DE FIEVRE ? POURQUOI VIENT-0N**

**CHEZ VOUS ? QUE FAITES-VOUS QUAND VOUS PENSEZ QUE LE CAS NE CONCERNE PAS VOS COMPETENCES ? LE REFEREZ-VOUS AU CENTRE DE SANTE ? PENDANT COMBIEN DE TEMPS LE GARDEZ-VOUS CHEZ VOUS AVANT LA REFERENCE ? RECEVEZ-VOUS CHEZ-VOUS DES REFERENCES DU PERSONNEL DU SANTE ?**

PROBE :1.3.4. WHEN DO PEOPLE CONSULT YOU FOR FEVER? WHY DO PEOPLE CONSULT YOU?

WHAT DO YOU DO WHEN THE CASE IS NOT FOR YOU ? DO YOU REFER THE PERSON TO THE HEALTH CENTER ? HOW LONG DO YOU KEEP THE PERSON IN YOUR PERISTYLE BEFORE THE REFERENCE ? DO YOU RECEIVE SOME REFERENCES FROM THE HEALTH PERSONNEL ?

**NOTES : LA SUITE DES QUESTIONS A POSER DEPENDRA DE LA CATEGORIE D’ INTERVIEWES DES REPONSES OBTENUES .**

THE LINES OF QUESTIONS WILL BE COMPLETED AS NEEDED.

**TOUT LE MONDE**

**FOR EVERY INTERVIEWEE**

**SECTION 2 : PERCEPTIONS DES STRATEGIES CLES TPE**

SECTION 2: TPE KEY STRATEGIES PERCEPTIONS

**2.1. AVEZ-VOUS DEJA RENCONTRÉ DES CAS DE MALARIA ?**

2.1. HAVE YOU ALREADY ENCOUNTERED PEOPLE WITH MALARIA ?

**A INVESTIGUER : 2.1.2. AVEZ-VOUS DEJA ENTENDU PARLER DU SNEM ?**

PROBE :2.1.2. HAVE YOU EVER HEARD ABOUT THE SNEM PROGRAM?

**A INVESTIGUER : 2.1.3. QUELLES ETAIENT LES ACTIVITES REALISEES PAR SLE SNEM DANS LES COMMUNAUTES ?**

PROBE : 2.1.3. WHAT WHERE SOME OF COMMUNITY ACTIVITIES REALIZED BY SNEM.

**SECTION 2: KEY STRATEGIES PERCEPTIONS TPE**

| *L’une des interventions essentielles de ce nouveau projet d’élimination du microbe de la malaria en Haïti est la distribution massive des antis malariques aux membres des communautés à haut risque pour la malaria. Il est prouvé que le microbe de la malaria peut être présent dans le sang des personnes bien portantes sans signes de maladie et parfois ne peut même pas être détecté par le laboratoire. C’est pourquoi il est nécessaire de donner des anti- malariques à toutes les personnes à haut risque. Elles ne seront pas testées avant la prise des médicaments. C’est ce que faisait le SNEM au cours des décennies 60, 70 et 80 du siècle dernier et c’est ce que fait actuellement le MSPP dans le programme contre la filariose ou encore appelee la maladie ‘filaryoz’ ou ‘Gwopye’.*  *Nous aimerions recueillir les opinions de la population sur certains aspects du programme de lutte contre la malaria en cours. Un des plus importants aspects de ce programme est d'offrir des médicaments aux personnes à haut risque dans les communautés pour le paludisme. La distribution se fera par un agent de santé de porte-à-porte ou au cours de réunions des gens de la communauté dans des endroits bien précis comme dans une école. Dans certains cas, le personnel de santé pourra offrir des tests de malaria et des médicaments aux personnes pour qui le test est positif afin de traiter le paludisme. Dans d'autres cas, le personnel de santé pourra distribuer des médicaments à tous dans la communauté même s'ils sont négatifs pour le paludisme car il est prouvé que les parasites sont présents, cachés dans l'organisme des membres de la population même sans produire des signes de maladie ou ne peuvent pas toujours être détectés par les tests de laboratoire. Il est essentiel que les membres de la communauté participent à ce genre de projets, et de prendre des médicaments contre le paludisme sans avoir les signes afin d'éliminer ce problème de santé en Haïti. Ceci est appelé distribution massive de médicaments contre le paludisme dans le cadre du projet : « Zero Malaria en Haiti  ».*  *One potential intervention of the malaria elimination project in Haiti is the mass distribution of anti malarial drugs to all people in communities at higher risk for malaria. It is proven that the parasites may be present, hidden in the body of the members of the population without any signs of disease or cannot always be detected by the laboratory. That is why it may be necessary to give all people in higher risk communities anti malarial medicine. They may not be tested before offering the drugs. This strategy is similar to what has been done by SNEM in the past programs for malaria also called in creole “maladi filaryoz or maladi Gwo pie.” It is known as “traitement de masse.” In french.*  *We would like to gather the community opinions on certain aspects of such a program The distribution may be done from door-to-door or at meetings of community people in specific places like in schools. In some cases, health workers can provide malaria tests and drugs to people. In other cases, health workers can dispense drugs to all in the community even if they are negative for malaria. It is essential that community members participate in such projects, and to take medication against malaria to eliminate this health problem in Haiti* |
| --- |

**2.2. DISTIBUTION MASSIVE DE MEDICAMENTS ANTI MALARIQUES**

2.2. MASS ANTI MALARIAL DRUG DISTRIBUTION

**A INVESTIGUER : 2.2.1. QUE PENSEZ-VOUS DE LA DISTRIBUTION MASSIVE DES MEDICAMENTS**

**ANTIMALARIQUES ?**

PROBE : 2.2.1. WHAT DO YOU THINK ABOUT MASS DRUG DISTRIBUTION TO TREAT SOME INFECTIOUS DISEASES SUCH AS MALARIA FOR EXAMPLE?

**A INVESTIGUER : 2.2.2. SOUTIENDREZ-VOUS UN TEL PROGRAMME DANS LA COMMUNAUTE?**

PROBE : 2.2.9. WILL YOU SUPPORT SUCH A PROGRAMM IN THE COMMUNITY ?

**A INVESTIGUER : 2.2.3. PENSEZ-VOUS QUE LA PLUPART DES GENS DANS LA COMMUNAUTE**

**ACCEPTERAIT DE PRENDRE DES MEDICAMENTS ANTIPALUDIQUES S’ILS SONT DISTRIBUÉS GRATUITEMENT ?**

PROBE 2.2.3. DO YOU THINK THAT MOST OF THE MEMBERS OF THE COMMUNITY WILL ACCEPT

TO TAKE THE ANTI MALARIAL DRUGS IF THEY ARE FREELY DISTRIBUTED?

**A INVESTIGUER : 2.2.4. EST-CE QUE LA PLUPART DES MEMBRES DE LA COMMUNAUTE ACCEPTERAIT**

**DE PRENDRE LES MEDICAMENTS SANS ETRE TESTÉS ET POURQUOI ?**

PROBE : 2.2.4. DO MOST OF THE COMMUNITY MEMBERS ACCEPT TO TAKE THE ANTIMALARIAL

DRUGS WITHOUT BEING TESTED AND WHY?

**A INVESTIGUER : 2.2.5. QUELS SONT LES DEFIS QU’AURONT À CONFONTER LA POPULATION POUR**

**PRENDRE LES ANTI MALARIQUES ? COMMENT RELEVER CES DEFIS ?**

PROBE : 2.2.5. WHAT ARE THE CHALLENGES THAT WOULD FACE THE POPULATION RELATED TO

ANTIMALARIAL DRUGS TAKING? HOW TO MEET THESE CHALLENGES?

**A INVESTIGUER : 2.2.6. QUI SONT CEUX QUI REFUSERAIENT D’EN PRENDRE SANS ETRE TESTÉS ?**

PROBE : 2.2.6. WHO ARE THOSE WHO WOULD REFUSE TO TAKE THEM WITHOUT BEING TESTED?

**INVESTIGUER : 2.2.7. COMMENT ATTEINDRE CEUX QUI REFUSERAIENT D’EN PRENDRE ET COMMENT**

**LES CONVAINCRE ?**

PROBE : 2.2.7. HOW TO REACH THOSE PEOPLE WHO WOULD REFUSE TO TAKE THE DRUGS AND

HOW TO CONVINCE THEM?

**A INVESTIGUER : 2.2.8. QUELLE EST LA MEILLEURE FACON DE DISTRIBUER LES ANTI PALUDIQUES ?**

**OÙ RENCONTRER LES GENS DE LA COMMUNAUTE : CHEZ EUX ? AU CENTRE**

**DE SANTÉ ? À UN AUTRE ENDROIT ? OÙ ?**

PROBE : 2.2.8. WHAT IS THE BEST WAY TO DISTRIBUTE ANTI MALARIAL DRUGS AND WHERE

TO MEET THOSE PEOPLE IN THE COMMUNITY: AT HOME? IN THE HEALTH CENTER? SOME OTHER PLACES ? WHERE ELSE?

**2.3. DEPISTAGE DE LA MALARIA**

2.3 . MALARIA SCREENING

NOTES : LE TEST DE MALARIA SE FAIT AU CENTRE DE SANTE PAR LE PERSONNEL POUR LES PATIENTS FREQUENTANT LE CENTRE DE SANTE.

**A INVESTIGUER : 2.3.1. QUE PENSEZ-VOUS DU TEST DE DEPISTAGE DE LA MALARIA AU CENTRE ?**

PROBE : 2.3.1. WHAT DO YOU THINK ABOUT MALARIA TESTING IN THE HEALTH CENTER?

**A INVESTIGUER : 2.3.2. SOUTIENDREZ-VOUS UNE TELLE PROCEDURE ?**

PROBE : 2.3.2. WILL YOU SUPPORT SUCH A PROCEDURE TO PROVIDE MALARIA DRUGS ?

**A INVESTIGUER : 2.3.3. QUELS SONT LES DEFIS QUE VA CONFONTER LA POPULATION POUR SE FAIRE**

**TESTER POUR MALARIA ?**

PROBE : 2.3.3. WHAT ARE THE CHALLENGES FACING THE POPULATION IN ORDER TO GET

TESTED FOR MALARIA ?

**A INVESTIGUER : 2.3.4. SI QUELQU’UN EST TESTÉ POSITIF, EST-CE QU’IL ACCEPTERAIT DE DONNER**

**SON NUMERO DE TÉLÉPHONE POUR QUE L’ON PUISSE MIEUX TRACER LA PROPAGATION DE LA MALARIA EN HAITI ?**

**NOTES : LE MODERATEUR EXPLIQUERA QUE LA CONFIDENTIALITÉ DE LA PERSONNE SERA RESPECTÉE ?**

PROBE : 2.3.4. IF SOMEONE IS TESTED POSITIVE, WOULD HE AGREES TO GIVE US HIS

CELL PHONE NUMBER IN ORDER FOR US TO LOCATE WHERE HE GOES? IF HIS CONFIDENTIALITY IS RESPECTED ?

NOTES : THE MODERATOR WILL EXPLAIN THAT THE MALARIA PROJECT WILL RESPECT THE CONFIDENTIALITY OF THE PERSON.

**A INVESTIGUER : 2.3.5. SOUTIENDREZ-VOUS UNE TELLE ACTIVITÉ ?**

PROBE : 2.3.5. WILL YOU SUPPORT SUCH AN ACTIVITY ?

**2.4. GROSSESSE ET MALARIA**

2.4. PREGNANCY AND MALARIA

**UN TEST DE GROSSESSE SERA OFFERT AUX FEMMES EN AGE DE PROCREER AFIN DE S’ ASSURER QUE DES ANTIMALARIQUES CONTREINDIQUES NE SOIENT ADMINISTRES A DES FEMMES ENCEINTES.**

**A PREGNANCY TEST WILL BE OFFERED TO ALL WOMEN AGED BETWEEN 15 AND 45 YEARS TO BE SURE THEY CAN HAVE CERTAIN ANTIMALARIAL DRUGS.**

**A INVESTIGUERIUER : 2.4.1. QUI SONT LES FEMMES QUI VONT ACCEPTER DE SE FAIRE TESTER POUR UNE GROSSESSE AVANT DE PRENDRE DES ANTI PALUDIQUES ?**

PROBE : 2.4.1. WHO ARE THE WOMEN WHO WILL ACCEPT TO GET TESTED FOR PREGNANCY

BEFORE TAKING ANTI MALARIAL DRUGS?

**A INVESTIGUER : 2.4.2. POURQUOI CES FEMMES VONT-ELLES ACCEPTER DE SE FAIRE TESTER ?**

PROBE : 2.4.2.2. WHY THESE WOMEN WILL ACCEPT TO GET TESTED ?

**A INVESTIGUER : 2.4.3. QUI SONT LES FEMMES QUI VONT REFUSER DE SE FAIRE TESTER POUR UNE**

**GROSSESSE AVANT DE PRENDRE DES ANTI PALUDIQUES ?**

PROBE : 2.4.3. WHO ARE THE WOMEN WHO WILL REFUSE TO GET TESTED FOR PREGNANCY

BEFORE OF ANTI MALARIA?

**A INVESTIGUER : 2.4.4. POURQUOI CES FEMMES REFUSERAIENT-ELLES DE SE FAIRE TESTER ?**

PROBE : 2.4.4. WHY THESE WOMEN WOULD REFUSE TO GET TESTED?

**A INVESTIGUER : 2.4.5. COMMENT ATTEINDRE ET CONVAINCRE CES FEMMES QUI REFUSENT DE SE**

**FAIRE TESTER ?**

PROBE : 2.4.5. HOW TO REACH AND CONVINCE THOSE WOMEN WHO REFUSE TO GET TESTED?

**A INVESTIGUER : 2.4.6. OÙ PROPOSERIEZ-VOUS DE TESTER CES FEMMES QUI REFUSENT INITIALEMENT DE SE FAIRE TESTER ?**

PROBE : 2.4.6. WHERE DO YOU PROPOSE TO TEST THOSE WOMEN WHO INITIALLY REFUSE TO

GET TESTED ?

**2.5. INFLUENCE SOCIALE**

**2.5. SOCIAL INFLUENCE**

**2.5.1. LA PRISE DE DECISION POUR LA PARTICIPATION AU TPE**

**2.5.1.1. QUI PEUT INFLUENCER LES GENS DE LA COMMUNAUTE A PARTICIPER A LA DISTRIBUTION MASSIVE DE MEDICAMENTS ?**

**A EXPLORER 2.5.1.1.1. LES ENSEIGNANTS, LES ECOLIERS, LES CHAUFFEURS D'AUTOBUS DANS**

**CETTE COMMUNAUTE ? QUI D’APRES VOUS INFLUENCENT LES GENS**

**DANS LA RECHERCHE DES SOINS DE SANTE EN GENERAL?**

PROBE 2.5.1.1. : SCHOOL TEACHERS, STUDENTS, BUS DRIVERS ? WHO CAN INFLUENCE

PEOPLE IN THE COMMUNITY IN ORDER TO GET HELP ?

**A EXPLORER 2.5.1.1.2. LES PROPRIETAIRES DE MAGASINS, ETC. OU DES GROUPES DE**

**PERSONNES**

**PROBE 2.5.1.1**.2. BUSINESS OWNERS, GROUPS OF PEOPLE ?

**NOTES : LES REPONSES DOIVENT D’ ABORD VENIR DES PARTICIPANTS D’UNE FAÇON SPONTANEE.**

**A EXPLORER 2.5.1.1.2. QUI INFLUENCENT LES GENS DE CETTE COMMUNAUTE POUR PRENDRE**

**DES MESURES DE PREVENTION DU PALUDISME, POUR SE FAIRE TESTER, ET POUR PRENDRE UN TRAITEMENT?**

PROBE : 2.5.1.1.2. WHO CAN INFLUENCE PEOPLE IN THE COMMUNITY TO PREVENT MALARIA,

TO GET TESTED AND TREATED

**A EXPLORER 2.5.1.1.3. EST-CE QUE LEUR INFLUENCE EST POSITIVE OU NEGATIVE?**

PROBE : 2.5.1.1.3. IS THEIR INFLUENCE POSITIVE OR NEGATIVE ?

**A EXPLORER 2.5.1.1.4. DE CES PERSONNES OU GROUPES DE PERSONNES QUE VOUS VENEZ**

**D’ENUMERER, COMMENT PEUT-ON LES CLASSER, DU PLUS FORT AU PLUS FAIBLE DE LEUR INFLUENCE, SUR LA PRISE DE DECISION CONCERNANT**

**LA MALARIA?**

PROBE : 2.5.1.1.4. HOW TO CLASSIFY THE INFLUENCE OF THE CITED PERSONS OR GROUPS

FROM THE WEAKEST TO THE STRONGEST ON DECISION MAKING ?

**A EXPLORER 2.5.1.1.5. QUELS SONT LES DIFFERENTS GROUPES ET** ORGANISATIONS **QUI**

**TRAVAILLENT DANS LA COMMUNAUTE ?**

PROBE : 2.5.1.1.5. WHAT ARE THE ORGANIZATIONS AND GROUPS OF PEOPLE WHO WORK IN

THE COMMUNITY ?

**A EXPLORER 2.5.1.1.6. QUI CONSTITUENT CES GROUPES? : HOMMES / FEMMES, LES VIEUX**

**/ JEUNES, OU AUTRES**

PROBE : 2.5.1.1.6. WHO CONSTITUTE THESE GROUPS ?MEN/WOMEN ? YOUNG/OLD? WHO ELSE?

**A EXPLORER 2.5.1.1.7. QUE FONT-ILS EN PARTICULIER OU EN GROUPE ?**

PROBE : 2.5.1.1.7. WHAT DO THESE GROUPS DO IN PARTICULAR OR IN THE COMMUNITY ?

**A EXPLORER 2.5.1.1.8. DE TOUS CES GROUPES ET ORGANISATIONS, QUELS SONT CEUX QUI**

**INFLUENCENT LES GENS DE LA COMMUNAUTE A CHERCHER DES SOINS DESANTE? COMMENT LE FONT-ILS?**

A EXPLORER 2.5.1.1.8.FROM ALL THOSE GROUPS AND ORGANISATIONS WHO ARE THOSE WHO

CAN INFLUENCE PEOPLE IN THE COMMUNITY TO SEEK HELP ? HOW CAN THEY DOIT ?

**2.5.1.2. MAINTENANT AU NIVEAU DES MENAGES, QUI INFLUENCENT LES GENS POUR CHERCHER**

**DES SOINS DE SANTE EN GENERAL?**

**2.5.1.2. AT THE HOUSEHOLD LEVEL, WHO CAN INFLUENCE THE MEMBERS OF THE FAMILY ?**

**A EXPLORER 2.5.1.2.1. COMMENT SONT PRISES DANS LES MENAGES LES DECISIONS**

**CONCERNANT LES SOINS DE SANTE ?**

A EXPLORER 2.5.1.2.1. HOW THE DECISONS ARE MADE IN THE HOUSEHOLDS ABOUT

HEALTH CARE ?

**A EXPLORER 2.5.1.2.2. COMMENT SONT PRISES LES DECISIONS CONCERNANT SPECIALEMENT LA**

**MALARIA DANS LES MENAGES ?**

A EXPLORER 2.5.1.2.2. HOW AND BY WHO THE DECISONS ARE MADE IN THE HOUSEHOLDS ABOUT MALARIA IN PARTICULAR?

**A EXPLORER 2.5.1.2.3. CONCERNANT LES STRATEGIES DE PREVENTION DE LA MALARIA COMME**

**LES PULVERISATIONS ET L’UTILISATION DE MOUSTIQUAIRES?**

A EXPLORER 2.5.1.2.3. BY WHOM THE DECISONS ARE MADE IN THE HOUSEHOLDS ABOUT

PREVENTION STRATEGIES LIKE PULVERIZATION AND BED NET

USE ?

**3. FERMETURE**

6. CLOSURE

**6.1. TOUJOURS EN RAPPORT AVEC CE DONT NOUS AVONS PARLE AUJOURD’HUI, AURIEZ-VOUS**

**D'AUTRES SUGGESTIONS POUR LE PROJET DE LA MALARIA?**

6.1. ACCORDING TO WHAT WE TALKED ABOUT TODAY, WOULD YOU HAVE OTHER SUGGESTIONS

CONCERNING THE MALARIA PROJECT?.

**6.2. AVEZ-VOUS DES SUGGESTIONS SUR D'AUTRES MOYENS D'AMELIORER L'ETUDE ?**

6.2 . DO YOU HAVE ANY SUGGESTIONS ON OTHER WAYS TO IMPROVE THE STUDY?

**6.3. NOUS ALLONS EGALEMENT PARLER DE CETTE ETUDE A D’ AUTRES PERSONNES DANS**

**CETTE COMMUNAUTE. NOUS AIMERIONS VOUS DEMANDER SI VOUS AVEZ DES PERSONNES A NOUS RECOMMANDER POUR CES DISCUSSIONS, ET POUR NOUS AIDER A LES CONTACTER.**

6.3. WE WILL TALK ALSO ABOUT THIS STUDY TO OTHER PEOPLE IN EACHCOMMUNITY .

WE WOULD LIKE TO ASK EACH OF YOU IF YOU HAVE PEOPLE YOU COULD RECOMMEND FOR THESE DISCUSSIONS, AND HOW YOU COULD HELP US TO GET IN TOUCH WITH THEM.

**MERCI DE PARTAGERS VOTRE TEMPS ET VOTRE EXPERTISE AVEC NOUS AUJOURD'HUI.**

THANK YOU FOR SHARING YOUR TIME AND YOUR EXPERTISE WITH US TODAY .
