# Supplementary material for: “Wherever doctors cannot reach, the sunshine can”: overcoming potential barriers to malaria elimination interventions in Haiti
Source: Malar J. 2018 Oct 29;17:393. doi: 10.1186/s12936-018-2553-5 (PMC6206681; doi:10.1186/s12936-018-2553-5)
Supplement: Supplementary file 3 — Additional file 3. Guide for focus group discussions. [file 12936_2018_2553_MOESM3_ESM.doc]

**MODÈLE GÉNÉRAL DE GUIDE DE GROUPE DE DISCUSSIONS POUR ASCP / LEADERS**

GENERAL MODEL OF THE FOCUS GROUP DISCUSSION GUIDE FOR ASCP / LEADERS

**LES ÉTAPES DE L’ENTREVUE**

**THE STAGES OF THE INTERVIEW**

| **Etape 1. Présenter l'équipe de recherche : Modérateurs, preneurs de notes**  Step 1. Introduce the research team: Moderators and Note takers  **Etape 2. Présenter le sujet de recherche: Je vous remercie d'être venus à notre groupe de discussion sur le paludisme. Nous vous remercions d'être ici avec nous. Nous menons ce groupe de discussion pour apprendre de votre expérience en tant que fournisseur de santé. Nous sommes particulièrement intéressés à savoir plus au sujet de la communauté que vous servez et de vos expériences concernant le paludisme.**  Step 2. Present the research topic: Thank you for coming to our panel discussion on malaria. Thank you for being here with us. We conduct this interview to learn from your experience on malaria in your community. We are particularly interested to learn more about the community and your experiences on malaria as Health Agents.  **Etape 3. Règles du jeu : Avant de commencer, permettez-moi de passer en revue quelques notions de base sur la façon dont le groupe va travailler. Il est important de se rappeler qu'il n'y a pas de bonnes ou de mauvaises réponses aux questions posées. Chaque personne peut avoir des opinions différentes sur certains de ces sujets. Même si ce que vous avez à dire est différent de ce que les autres dans le groupe pensent, nous voulons l'entendre. Nous voulons entendre ce que tout le monde pense. En outre, la discussion de groupe sera menée sous forme d'une conversation tout en respectant les règles suivantes: Chacun des participants a la chance de s'exprimer en respectant le droit d'expression des autres. Les participants ne sont pas obliges d'être d'accord ou en désaccord avec les autres suivant le cas et doivent présenter leurs arguments pour appuyer leur point de vue. Les participants peuvent prendre la parole un seul à la fois après avoir obtenu la permission du modérateur et parler a haute et intelligible voix pour nous permettre d' entendre clairement et enregistrer ce que vous dites. Et enfin rappelez-vous que tous les commentaires ou opinions exprimés par les participants a ce groupe de discussion sont considérés comme strictement confidentiels et nous vous demandons de ne pas les partager ou d'en discuter en dehors de ce groupe en laissant la table de réunion.**  **Le modérateur demande ensuite aux participants s'ils auraient des questions ou un point à éclaircir avant le début de la discussion.**  Step 3. Rules: Before we start, let me review some basics principles about how we will work. It is important to remember that there are no right or wrong answers to the questions. Each person can have different opinions on some of these subjects. Even if what you say is different from what others in the group think or say we want to hear it. We want to hear what everyone thinks. In addition, the focus group will be conducted as a conversation with the following rules: Each participant has the opportunity to speak while respecting the right of expression of other ones. Participants are not obliged to agree or disagree with others as appropriate and must present their arguments to support their views. Participants can speak one at a time after obtaining the permission of the moderator and talk in a loud and clear voice to allow us to clearly hear and record what you say.  And finally remember that any comments or opinions expressed by participants in this focus group are considered strictly confidential and we ask you not to share or discuss it outside this group after leaving the meeting table. The moderator then ask the participants if they have questions or point to clarify them before the start of the discussion.  **Etape 4. Signature du consentement informé**  Step 4. Signature of informed consent  **Etape 5. Identification des participants - Remise des badges**  Step 5. Identification of the participants - Badges  **Etape 6. Collecte des données démographiques**  Step 6. Demographic data collection  **Etape 7. Entrevue**  Srep 7. Interview |
| --- |

**SECTION 1. PRESENTATION (5 - 10 minutes)**

**1.1. PRESENTATION DES MODERATEUR ET PRENEUR DE NOTES**

1.1. PRESENTATION OF MODERATOR AND NOTES TAKER

**1.2. PRESENTATION DES PARTICIPANTS**

1.2. PRESENTATION OF PARTICIPANTS

**1.3. POSITIONNEMENT DES PARTICIPANTS ET DONNEES DEMOGRAPHIQUES**

1.3. POSITIONING OF PARTICIPANTS AND DEMOGRAPHICS

**SECTION 2 : PERCEPTIONS DES STRATEGIES CLES TPE**

SECTION 2: TPE KEY STRATEGIES PERCEPTIONS

**2.1. CONNAISANCE DE LA MALARIA ?**

2.1. KWOWLEDGE ABOUT MALARIA ?

**A INVESTIGUER : 2.1.1. QUE SAVEZ-VOUS OU AVEZ –VOUS ENTENDU PARLER DE LA MALARIA ? CONNAISSEZ-VOUS DES GENS DE LA COMMUNAUTE QUI ONT DEJA EU LA MALARIA ? SI OUI, QU’AVAIENT-ILS FAIT POUR AVOIR DE L’AIDE ? COMMENT AURAIENT-ILS PU EVITER D’ATTRAPER LA MALADIE ?**

PROBE : 2.1.1. WHAT DO YOU KNOW ABOUT MALARIA ? DO YOU KNOW PEOPLE IN YOUR COMMUNITY WHO HAVE HAD MALARIA? IF SO WHAT THOSE PEOPLE DID TO GET CARE FOR THAT DISEASE? COMMENT POURRAIT –ON EVITER D’ ATTRAPER LA MALARIA ?

**A INVESTIGUER : 2.1.2. AVEZ-VOUS DEJA ENTENDU PARLER DU SNEM ?**

PROBE :2.1.2. HAVE YOU EVER HEARD ABOUT THE SNEM PROGRAM?

**A INVESTIGUER : 2.1.3. QUE FAISAIT LE SNEM DANS LA ZONE ?**

PROBE : 2.1.3. WHAT HAVE SNEM DONE IN THE AREA

**SECTION 2: TPE KEY STRATEGIES PERCEPTIONS**

| *L’une des interventions essentielles de ce nouveau projet d’élimination du microbe de la malaria en Haïti est la distribution massive des antis malariques aux membres des communautés à haut risque pour la malaria. Il est prouvé que le microbe de la malaria peut être présent dans le sang des personnes bien portantes sans signes de maladie et parfois ne peut même pas être détecté par le laboratoire. C’est pourquoi il est nécessaire de donner des anti malariques à toutes les personnes à haut risque. Elles ne seront pas testées avant la prise des médicaments. C’est ce que faisait le SNEM au cours des décennies 60, 70 et 80 du siècle dernier et c’est ce que fait actuellement le MSPP dans le programme contre la filariose ou encore contre la maladie ‘filaryoz’ ou ‘Gwopye’.*  *Nous aimerions recueillir les opinions de la population sur certains aspects du programme de lutte contre la malaria en cours. Un des plus importants aspects de ce programme est d'offrir des médicaments aux personnes à haut risque dans les communautés pour le paludisme. La distribution se fera par un agent de santé de porte-à-porte ou au cours de réunions des gens de la communauté dans des endroits bien précis comme dans une école. Dans certains cas, le personnel de santé pourra offrir des tests de malaria et des médicaments aux personnes pour qui le test est positif afin de traiter le paludisme. Dans d'autres cas, le personnel de santé pourra distribuer des médicaments à tous dans la communauté même s'ils sont négatifs pour le paludisme car il est prouvé que les parasites sont parfois présents, cachés dans l'organisme des membres de la population même sans produire des signes de maladie ou ne peuvent pas toujours être détectés par les tests de laboratoire. Il est essentiel que les membres de la communauté participent à ce genre de projets, et de prendre des médicaments contre le paludisme sans avoir les signes afin d'éliminer ce problème de santé en Haïti. Ceci est appelé distribution massive de médicaments contre le paludisme.*  *One potential intervention of the malaria elimination project in Haiti is the mass distribution of anti malarial drugs to all people in communities at higher risk for malaria. It is proven that the parasites may be present, hidden in the body of the members of the population without any signs of disease or cannot always be detected by the laboratory. That is why it may be necessary to give all people in higher risk communities anti malarial medicine. They may not be tested before offering the drugs. This strategy is similar to what has been done by SNEM in the past and for programs for “maladi filaryoz or maladi Gwo pie.” It is known as “tretman de mas.”*  *We would like to gather information on on certain aspects of such a program The distribution may be done from door-to-door or at meetings of community people in specific places like in schools. In some cases, health workers can provide malaria tests and drugs to people. In other cases, health workers can dispense drugs to all in the community even if they are negative for malaria. It is essential that community members participate in such projects, and to take medication against malaria to eliminate this health problem in Haiti* |
| --- |

**2.2. DISTIBUTION MASSIVE DE MEDICAMENTS ANTI MALARIQUES**

2.2. MASS DRUG DISTRIBUTION

**A INVESTIGUER : 2.2.1. QUE PENSEZ-VOUS DE LA DISTRIBUTION MASSIVE DES MEDICAMENTS**

**ANTIMALARIQUES ?**

PROBE : 2.2.1. WHAT DO YOU THINK ABOUT MASS DRUG DISTRIBUTION TO TREAT SOME INFECTIOUS DISEASES SUCH AS MALARIA FOR EXAMPLE?

**A INVESTIGUER : 2.2.2. PENSEZ-VOUS QUE LA PLUPART DES GENS DANS LA COMMUNAUTE**

**ACCEPTERAIT DE PRENDRE DES MEDICAMENTS ANTIPALUDIQUES S’ILS SONT DISTRIBUÉS GRATUITEMENT ?**

PROBE 2.2.2. DO YOU THINK THAT MOST OF THE MEMBERS OF THE COMMUNITY WILL ACCEPT

TO TAKE THE ANTI MALARIAL DRUGS IF THEY ARE FREELY DISTRIBUTED?

**A INVESTIGUER : 2.2.3. EST-CE QUE LA PLUPART DES MEMBRES DE LA COMMUNAUTE ACCEPTERAIT**

**DE PRENDRE LES MEDICAMENTS SANS ETRE TESTÉS ET POURQUOI ?**

PROBE : 2.2.3. DO MOST OF THE COMMUNITY MEMBERS ACCEPT TO TAKE THE ANTIMALARIAL

DRUGS WITHOUT BEING TESTED AND WHY?

**A INVESTIGUER : 2.2.4. EST-CE QUE LA PLUPART DES MEMBRES DE LA COMMUNAUTE ACCEPTERAIT**

**DE PRENDRE LES MEDICAMENTS S’ILS SONT TESTÉS POSITIFS ?**

PROBE : 2.2.4. WOULD MOST OF THE COMMUNITY MEMBERS WOULD ACCEPT TO TAKE THE

MEDICATIONS IF THEY ARE TESTED POSITIVE?

**A INVESTIGUER : 2.2.5. Y A-IL CERTAINS GROUPES DE PERSONNES QUI ACCEPTERAIENT DE**

**PRENDRE LES MEDICAMENTS ANTI MALARIQUES ?**

PROBE : 2.2.5. ARE THERE CERTAIN GROUPS OF PEOPLE THAT WOULD ACCEPT TO TAKE THE

ANTIMALARIAL DRUGS?

**A INVESTIGUER : 2.2.7. QUI SONT CEUX QUI REFUSERAIENT D’EN PRENDRE SANS ETRE TESTÉS ?**

PROBE : 2.2.7. WHO ARE THOSE WHO WILL REFUSE TO TAKE THEM WHITHOUT BEING

TESTED ?

**A INVESTIGUER : 2.2.6. QUELS SONT LES DEFIS QU’AURONT À CONFONTER LA POPULATION POUR**

**PRENDRE LES ANTI MALARIQUES ? COMMENT RELEVER CES DEFIS ?**

PROBE : 2.2.6. WHAT ARE THE CHALLENGES THAT WOULD FACE THE POPULATION RELTED TO

TO ANTIMALARIAL DRUGS TAKING? HOW TO MEET THESE CHALLENGES?

**A INVESTIGUER : 2.2.8. Y A-IL CERTAINS GROUPES DE PERSONNES QUI REFUSERAIENT DE PRENDRE**

**LES MEDICAMENTS ANTI MALARIQUES ?**

PROBE : 2.3.8. ARE THERE CERTAIN GROUPS OF PEOPLE WHO WOULD REFUSE TO TAKE THE

DRUGS / TEST ?

**A INVESTIGUER : 2.2.9. COMMENT ATTEINDRE CEUX QUI REFUSERAIENT D’EN PRENDRE ET COMMENT**

**LES CONVAINCRE ?**

PROBE : 2.2.9. HOW TO REACH THOSE PEOPLE WHO REFUSE TO TAKE THE DRUGS AND HOW

TO CONVINCE THEM ?

**A INVESTIGUER : 2.2.10. QUELLE EST LA MEILLEURE FACON DE DISTRIBUER LES ANTI PALUDIQUES ?**

**OÙ RENCONTRER LES GENS DE LA COMMUNAUTE : CHEZ EUX ? AU CENTRE**

**DE SANTE ? A UN AUTRE ENDROIT ? OÙ ?**

PROBE : 2.2.10. WHAT IS THE BEST WAY TO DISTRIBUTE ANTI MALARIAL DRUGS OR WHERE

TO MEET THOSE PEOPLE IN THE COMMUNITY: AT HOME? IN THE HEALTH CENTER ? SOME OTHER PLACES ? WHERE ELSE?

**2.3. DEPISTAGE DE LA MALARIA**

2.3 . MALARIA SCREENING

NOTES : LE TEST DE MALARIA SE FAIT AU CENTRE DE SANTE PAR LE PERSONNEL POUR LES PATIENTS FREQUENTANT LE CENTRE DE SANTE.

**A INVESTIGUER : 2.3.1. QUE PENSEZ-VOUS DU TEST DE DEPISTAGE DE LA MALARIA AU CENTRE ?**

PROBE : 2.3.1. WHAT DO YOU THINK ABOUT MALARIA TESTING IN THE HEALTH CENTER?

**A INVESTIGUER : 2.3.2. SOUTIENDREZ-VOUS UNE TELLE PROCEDURE ?**

PROBE : 2.3.2. WILL YOU SUPPORT SUCH A PROCEDURE TO PROVIDE MALARIA DRUGS ?

**A INVESTIGUER : 2.3.3. QUELS SONT LES DEFIS QUE VA CONFONTER LA POPULATION POUR SE FAIRE**

**TESTER POUR MALARIA ?**

PROBE : 2.3.3. WHAT ARE THE CHALLENGES FACING THE POPULATION IN ORDER TO GET

TESTED FOR MALARIA ?

**A INVESTIGUER : 2.3.4. SI QUELQU’UN EST TESTÉ POSITIF, EST-CE QU’IL ACCEPTERAIT DE DONNER**

**SON NUMERO DE TÉLÉPHONE POUR QUE L’ON PUISSE MIEUX TRACER LA PROPAGATION DE LA MALARIA EN HAITI ?**

**NOTES : LE MODERATEUR EXPLIQUERA QUE LA CONFIDENTIALITÉ DE LA PERSONNE SERA RESPECTÉE ?**

PROBE : 2.3.4. IF SOMEONE IS TESTED POSITIVE, WOULD HE AGREES TO GIVE US HIS

CELL PHONE NUMBER IN ORDER FOR US TO LOCATE WHERE HE GOES? IF HIS CONFIDENTIALITY IS RESPECTED ?

**A INVESTIGUER : 2.3.5. SOUTIENDREZ-VOUS UNE TELLE ACTIVITÉ ?**

PROBE : 2.3.5. WILL YOU SUPPORT SUCH AN ACTIVITY ?

**2.4. GROSSESSE ET MALARIA**

2.4. PREGNANCY AND MALARIA

**NOTES : CERTAINS MEDICAMENTS ANTI MALARIQUES SONT CONTRE INDIQUÉS POUR LES FEMMES EMCEINTES.**

**A INVESTIGUER : 2.4.3. QUI SONT LES FEMMES QUI VONT ACCEPTER DE SE FAIRE TESTER POUR UNE**

**GROSSESSE AVANT DE PRENDRE DES ANTI PALUDIQUES ?**

PROBE : 2.4.3. WHO ARE THE WOMEN WHO WILL ACCEPT TO GET TESTED FOR PREGNANCY

BEFORE TAKING ANTI MALARIAL DRUGS ?

**A INVESTIGUER : 2.4.4. POURQUOI CES FEMMES VONT-ELLES ACCEPTER DE SE FAIRE TESTER ?**

PROBE : 2.4.4. WHY THESE WOMEN WILL ACCEPT TO GET TESTED WOMEN?

**A INVESTIGUER : 2.4.5. QUI SONT LES FEMMES QUI VONT REFUSER DE SE FAIRE TESTER POUR UNE**

**GROSSESSE AVANT DE PRENDRE DES ANTI PALUDIQUES ?**

PROBE : 2.4.5. WHO ARE THE WOMEN WHO WILL REFUSE TO GET TESTED FOR PREGANCY

BEFORE HAVING ANTI MALARIA?

**A INVESTIGUER : 2.4.6. POURQUOI CES FEMMES REFUSERAIENT-ELLES DE SE FAIRE TESTER ?**

PROBE : 2.4.6. WHY THESE WOMEN WILL THEY REFUSE TO GET TESTED?

**A INVESTIGUER : 2.4.7. COMMENT ATTEINDRE ET CONVAINCRE CES FEMMES QUI REFUSENT DE SE**

**FAIRE TESTER ?**

PROBE : 2.4.7. HOW TO REACH AND CONVINCE THOSE WOMEN WHO REFUSE TO GET TESTED ?

**A INVESTIGUER : 2.4.8. OÙ PROPOSERIEZ-VOUS DE TESTER CES FEMMES QUI REFUSENT INITIALEMENT DE SE FAIRE TESTER ?**

PROBE : 2.4.8. WHERE DO YOU PROPOSE TO TEST FOR PREGNANCY THOSE WOMEN WHO

INITIALLY REFUSE TO GET TESTED ?

**3. FERMETURE**

3. CLOSURE

**3.1. TOUJOURS EN RAPORT AVEC CE DONT NOUS AVONS PARLE AUJOURD’HUI, AURIEZ-VOUS D'AUTRES SUGGESTIONS POUR LE PROJET DE LA MALARIA?**

3.1. ACCORDING TO WHAT WE TALKED ABOUT TODAY, WOULD YOU HAVE OTHER SUGGESTIONS CONCERNING THE MALARIA PROJECT?.

**3.2. AVEZ-VOUS DES SUGGESTIONS SUR D'AUTRES MOYENS D'AMELIORER L'ETUDE ?**

3.2 . DO YOU HAVE ANY SUGGESTIONS ON OTHER WAYS TO IMPROVE THE STUDY ?

**3.3. NOUS ALLONS EGALEMENT PARLER DE CETTE ETUDE A D’ AUTRES PERSONNES DANS CHAQUE COMMUNAUTE . NOUS AIMERIONS DEMANDER A CHACUN DE VOUS SI VOUS AVEZ DES PERSONNES A NOUS RECOMMANDER POUR CES DISCUSSIONS, ET POUR NOUS AIDER A LES CONTACTER.**

3.3. WE WILL TALK ALSO ABOUT THIS STUDY TO OTHER PEOPLE IN EACH COMMUNITY . WE WOULD LIKE TO ASK EACH OF YOU IF YOU HAVE PEOPLE YOU COULD RECOMMEND FOR THESE DISCUSSIONS , AND HOW YOU COULD HELP US TO GET IN TOUCH WITH THEM.

**MERCI DE PARTAGER VOTRE TEMPS ET VOTRE EXPERTISE AVEC NOUS AUJOURD'HUI.**

THANK YOU FOR SHARING YOUR TIME AND YOUR EXPERTISE WITH US TODAY .
